# Enabling Smart Workflows over Heterogeneous ID-Sensing Technologies

**DOI:** 10.3390/s121114914

**Published:** 2012-11-05

**Authors:** Pau Giner, Carlos Cetina, Raquel Lacuesta, Guillermo Palacios

**Affiliations:** 1 Pervasive Computing Group, San Jorge University, Autovia A-23 Zaragoza-Huesca Km. 299, Villanuevade Gllego (Zaragoza) 50830, Spain; E-Mail: pau.giner@gmail.com; 2 Department of Computing and Systems Engineering, University of Zaragoza, Ciudad Escolar s/n, Teruel 44003, Spain; E-Mails: lacuesta@unizar.es (R.L.); guillermo.palacios@unizar.es (G.P.)

**Keywords:** internet of things, smart workflows, mobile computing

## Abstract

Sensing technologies in mobile devices play a key role in reducing the gap between the physical and the digital world. The use of automatic identification capabilities can improve user participation in business processes where physical elements are involved (Smart Workflows). However, identifying all objects in the user surroundings does not automatically translate into meaningful services to the user. This work introduces Parkour, an architecture that allows the development of services that match the goals of each of the participants in a smart workflow. Parkour is based on a pluggable architecture that can be extended to provide support for new tasks and technologies. In order to facilitate the development of these plug-ins, tools that automate the development process are also provided. Several Parkour-based systems have been developed in order to validate the applicability of the proposal.

## 1. Introduction

The capabilities of mobile devices for sensing real-world elements in their surroundings (ID-sensing) are becoming widespread. Some examples of these capabilities are the use of on-board cameras for decoding 2D barcodes or the use of Near Field Communication (NFC) technology for reading RFID contactless tags. ID-sensing technologies alleviate the inherent I/O limitations of mobile devices by automating the linkage between physical and digital spaces.

When leveraging the ID-sensing capabilities of mobile devices, daily activities become more fluid for users. For example, users in a library can borrow a book just by taking a picture of its barcode with their mobile devices. Consequently, the information system of the library registers the loan and the alarm does not go off when the book is detected at the exit of the library. In this way, each user can avoid queues by managing his/her loans autonomously. By accessing the digital services associated with the book, users can also complement their reading with the opinions of others: users can post, rate and read comments about the book. Once the book is returned, the librarian can use an NFC-equipped mobile device to query where the book should be placed just by “touching” it with the device, and immediately the corresponding location is provided to the librarian.

This work is focused on providing support for Smart Workflows [1], which are business processes that cross the boundary to the physical world. The use of ID-sensing in this context has been demonstrated to be successful, reducing media breaks, human errors and delayed information problems [2]. Although smart workflows focus on process automation, a complete automation of business processes is not always possible or desirable [3]. Thus, user participation in business processes is still required. Considering the widespread availability of mobile devices, we propose their use to enable users to effectively participate in smart workflows.

Based on the principles for Smart Workflow design introduced in [4], this work introduces Parkour, an architecture and development process to support smart workflows across different devices. Parkour allows the user to connect smart workflow with the real world in different ways. On the one hand, Parkour provides context-aware task management. In other words, it indicates to users the tasks that they can do according to (1) their role in the process, (2) the physical environment and (3) the process state. For example, when a librarian (role of the user) selects a book (physical context) that has been returned (process state), interaction mechanisms that help the user to perform the “place the book on its shelf” task are provided. On the other hand, Parkour allows the use of the real world as the user interface for completing process tasks. The data demanded by a task can be provided by “touching” the physical object whose associated information is required.

The remainder of the paper is structured as follows. Section 2 presents the approach followed by Parkour for the support of smart workflows. Section 3 introduces the Parkour architecture. Section 4 provides some results from the use of Parkour in different case studies. Section 5 gives some insights about automation in the development of Parkour-based solutions. Related work is presented in Section 6. Finally, Section 7 presents conclusions and further work.

## 2. Supporting User Participation in Smart Workflows with Parkour

Most of the research in Ubiquitous Computing has been focused on supporting the informal and unstructured activities that are typical of much of our everyday lives [5]. However, the smart workflow application domain targeted in this work is different, since it is focused on well-defined and structured activities. Applications of this domain are characterized by a clear definition of the activities involved and their coordination. Workflow techniques promote methodologies and tools to support the modeling, execution, and management of business processes [6]. For example, the loan process of a library or the production method in a factory follows a defined procedure that is composed by a clear sequence of steps that must be followed.

When users participate in a workflow, they are under a constrained freedom. They can choose which tasks to perform, but they are limited in their decisions by the rules derived from the business process definition—e.g., a book can be returned by a member only if it has been borrowed. The freedom to choose a task can be due to the process definition (*i.e.*, the process provides an explicit choice for the user) or due to the user task management (*i.e.*, the user decides to postpone his action and moves to a different process instance from his “to do list”).

When physical objects are involved in multiple workflows, a different set of actions can be associated to them in each workflow. For example, the same physical book can be involved in two workflows such as “lending process” and “cataloguing”. In the lending process, the user is interested in borrowing the book. Conversely, in the cataloguing process the librarian is interested in locating the correct placement for a misplaced book. For each of the previous workflows, a given user needs different information and services about the same book.

Parkour is designed to offer at any time the functionality that users need for their participation in the smart workflow—that is, the information and actions about physical elements in the user surroundings that are relevant to the workflow. In order to achieve this, Parkour provides support to (1) general operation mechanisms that are useful for any smart workflow and (2) the possibility of customization in order to respond to the specific needs of a particular application. In the same way as Web browsers provide common concepts to the user regardless of the current web page being displayed—such as the back button or the add to bookmarks concept, Parkour provides some general operation modes and concepts regardless of the supported task in the smart workflow. Thus, users can become familiar with the use of the platform regardless of the task they are completing. In addition, given this common basis, the possibilities for customization remain high. The users—e.g., librarians, library members, maintenance technicians, *etc.*, can incorporate a set of plug-ins [7] to Parkour in order to turn the generic tool into a specific client that supports their particular perspective in the smart workflow.

Two challenges are faced by Parkour for supporting the multiple user perspectives of smart workflows in a flexible manner. The solution that our platform provides is what distinguishes it from other already available ID-sensing solutions, as is detailed below.

### 2.1. Extensibility of the Platform

Most of the available ID-sensing solutions for mobile devices such as QR Code readers are focused on supporting a single ID-sensing technology and they have no notion of current user activities. These clients normally provide access to information associated with a physical element—e.g., product price—regardless of the task in which the user is engaged.

In contrast to these solutions, our approach is capable of supporting multiple tasks and multiple ID-sensing technologies thanks to its pluggable nature. Plug-ins with a well-defined function-support for task—a technology or a data source—can extend the platform to specific requirements. For each task of the business process that a user is in charge of performing, the platform is extended with a plug-in that provides the adequate interaction mechanisms for completing this task. In addition, if a plug-in that supports a new ID-sensing technology is incorporated to the platform, any of the existing tasks in the platform can immediately take advantage of the new technology.

### 2.2. Flexibility in the Physical-Virtual Linkage

A common approach for augmenting physical elements with digital services is to include links to the digital services—e.g., URLs or e-mail addresses—in the tags that are attached to the physical elements. Since this information is embedded in the tag of the element, it is difficult to add, modify or customize services without altering this tag [8]. This coupling between identifiers and services can be assumed in many application contexts where the physical-virtual linkage is static, but it becomes a problem when dealing with smart workflows. In a smart workflow, physical elements are augmented with services provided by different actors—manufacturers, maintenance companies, retailers, *etc.*, and the users consuming them are interested only on a subset of these services that depends on the business process context—e.g., a service for placing a book into its shelf is interesting for librarians only when the book is returned, but not while they are engaged in other activities.

To confront this problem, our approach decouples the identification of elements from the services that are provided to each user. Tags are only used as identifiers for physical elements without including any reference to the services in which the element can participate. Parkour allows a physical element to be mapped with many digital services, and this mapping is dynamically filtered by Parkour in order to offer only the digital services that are relevant for the process state—*i.e.*, user role in the process and the activities in which they are engaged.

The following sections provide more detail on the Parkour platform and on how it supports user participation in smart workflows with regard to the extensibility of the platform and the flexibility in the physical-virtual linkage.

## 3. Elaboration of the Architecture

The architecture of Parkour has been defined following an architectural process introduced by Völter in [9]. This process is designed to obtain software architectures that are minimally affected by technological cycles and to permit an easy evolution of requirements by means of automation in the development. These properties are especially interesting in the case of Parkour considering that (1) the high heterogeneity in mobile platforms and ID-sensing technologies discourages the definition of the architecture in a tightly coupled manner with a particular technological platform; and (2) the fast-changing nature of business processes requires mechanisms to effectively project these changes into software solutions. Völter proposes an architectural process that is based on three general stages:

1. First, the elaboration phase of the architecture defines the architecture by decoupling the technology-independent concepts from the actual technological solutions.
2. The iteration phase serves to consolidate the architecture by receiving feedback from its use. The Parkour-based systems developed in this phase are introduced in Section 4.
3. Finally, the use of the architecture can be improved by avoiding repetitive programming tasks with automation. In this way, software development for the architecture becomes more efficient. The tools provided for automating the development are introduced in Section 5.

The present section introduces the elaboration phase of the architecture. According to the architectural process followed, the elaboration of the architecture defines the architecture first at a conceptual level. This constitutes a technology-independent architecture. Then, usage guidelines for the defined concepts are established in a programming model. The technology mapping defines how artifacts from the programming model are mapped to a particular technology. A mock platform facilitates testing tasks for developers. Finally, the development of a vertical prototype helps to evaluate the architecture and provides feedback about non-functional requirements.

### 3.1. Technology-Independent Architecture

In this section, we present a technology-independent description of the architecture introduced in this paper. By using a description based on technology-neutral concepts, we obtain a sustainable software architecture—*i.e.*, an architecture that is not affected by technological hypes and can evolve over time throughout several technological cycles.

For the definition of the architecture, we rely on the component concept since this is a well-understood concept that can be implemented in most of the implementation technologies available. Components are the basic software pieces that conform the system. Component functionality is described by means of interfaces, and the communication among components is performed asynchronously. We have opted for asynchronous communication since the business processes considered are usually long-running and involve human participation.

In the design of the architecture, it is assumed that external services are in control of the process flow. Services such as business process execution engines, calendar applications or personal task management software can require some task to be performed by the user. Our architecture is in charge of processing these demands and offers the appropriate mechanisms for the user to complete them making use of ID-sensing technologies.

**Figure 1.**
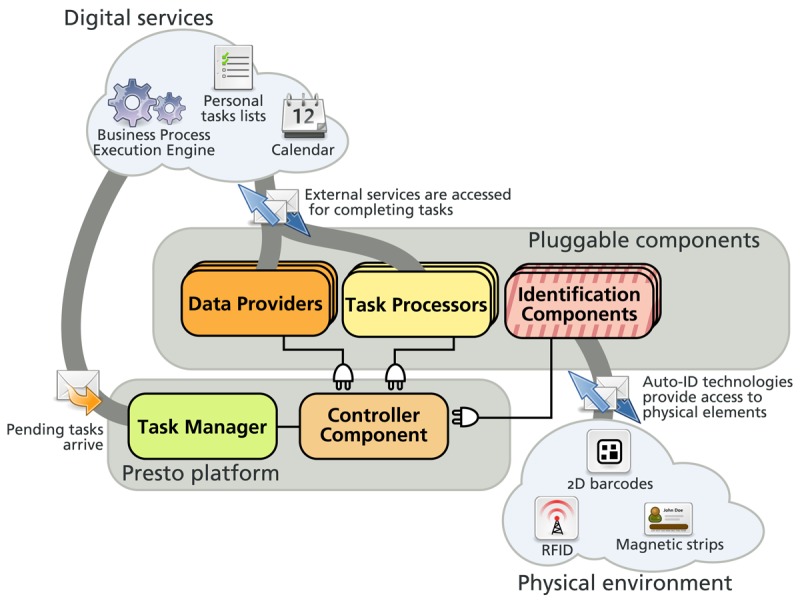
Architecture component overview.

The Parkour architecture is composed of a Task Manager, a Controller Component, and several Task Processors, Identification Components and Data Providers. Figure 1 illustrates the components involved in the Parkour architecture and their connection to digital and physical spaces. More detail about these components is given below.

#### 3.1.1. Task Manager

This component acts as a buffer where pieces of pending work are waiting to be completed. It receives messages from external services such as the information system of a library and waits for components to process them. The *Task Manager* can be queried in order to retrieve messages according to certain criteria—e.g., tasks of a certain type, involving a given physical element or targeted to a specific user.

The Task Manager handles messages of two kinds: pending tasks and notifications. Both kinds of messages provide information to the user, with the difference being that the pending tasks require some information to be provided as a response. This response message consists of (1) the confirmation that the task has been completed and, optionally, (2) additional information that is specific to the task. Conversely, notifications are announced to the user without requiring further interaction.

Task Manager has a central role in offering a flexible physical-virtual linkage since the information handled by this component—*i.e.*, the business process context—is used to determine the services to be offered to each user.

#### 3.1.2. Task Processors

These components are in charge of supporting the extensibility of the platform in terms of business functionality. A Task Processor is defined to handle a particular type of task—e.g., borrowing a book from the library as in the example. Task Processors provide the users with the required information, services and interaction mechanisms for completing the task. The interaction mechanisms provided are not limited to the classical display-driven user interfaces, since alternative user interfaces have proven to be successful for managing tasks with mobile devices [10]. In particular, Identification Components are used for accessing the physical environment. For example, the Task Processor for the borrow book task of the library example is in charge of retrieving the required information for the loan—member and book—which is sent to the digital services of the library in order to register this loan. In this case, the book information is retrieved from the physical world by means of an Identification Component.

#### 3.1.3. Identification Components

These components are in charge of supporting the extensibility of the platform at the technological level. Identification Components provide mechanisms for accessing the physical environment and transferring identifiers between physical and digital spaces by means of some ID-sensing technology. These components provide facilities for capturing and generating identifiers with a specific technology. In the library example, two Identification Components are used for the capture of identifiers; one for accessing RFID tags, which is deployed in the librarian mobile device, and another for 2D Barcode recognition, which is deployed in the devices of the library members.

#### 3.1.4. Data Providers

The Data Providers are in charge of transforming the identifiers provided by the Identification Components into information that is relevant for the user. The Data Providers are in charge of dynamically establishing the connection between the physical elements and their virtual counterparts. Thus, they are the basis for achieving a flexible physical-virtual linkage. Each Data Provider represents a possible projection of a physical element in the digital world. For example, a physical element such as a book can offer the access to the services provided by the library, the author who wrote it, or its publishers. Each of these perspectives is offered by a different Data Provider.

A Data Provider is in charge of (1) determining whether an identifier corresponds to an element of a certain kind, and (2) providing related information about this element. Digital services—e.g., EPC Information Services, product databases, *etc.*—can be accessed for both purposes, for checking that the identifier belongs to a list of elements of a certain kind and/or for retrieving related information. However, in some cases, the encoded information in the identifier allows the physical element to be classified without accessing external services—e.g., it includes manufacturer information.

#### 3.1.5. Controller Component

The controller is in charge of handling the extensibility of the platform by coordinating the above components. The Controller Component transfers the information among the different components in the architecture. The way in which information is transferred depends on the operation mode considered. Parkour supports two general operation modes: object-driven mode and task-driven mode.

In the object-driven mode, the physical context is sensed first and the related tasks are then proposed to the user. For example, a user touches a book with a device and the services associated with this book—such as borrowing it—are presented to the user. In this way, the user finds out what can be done in a given physical context. In the task-driven mode, the user explicitly indicates which task he/she is performing. The physical context is then accessed for completing this specific task. For example, the user decides to borrow a book and then he/she selects the book to be lent.

**Figure 2.**
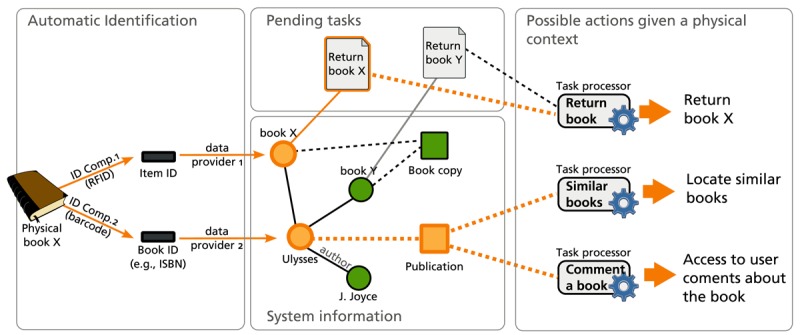
Example of the role of identification components, data providers and task processors.

When developing a particular system that follows our architecture, some components are generic and can be reused, while others must be developed for the particular domain. In the current architecture, the Task Manager and the Controller Component are generic; therefore, they can be used in any domain. The rest of the components—Task Processors, Identification Components and Data Providers—are defined as plug-ins for the architecture that can be added to customize the platform to the specific domain needs. Figure 2 illustrates how the introduced components interact when a physical element is detected. In the example, two identification components provide different identifiers, which are mapped by Data Providers into different digital counterparts. One data provider considers the physical element as a particular book copy—Book X—that can be borrowed at item level. The other data provider considers the physical book as a representation of a literary work to which copies such as Book X and Book Y belong. Each of these counterparts can be (1) part of a pending task generated by the business process execution engine, or (2) of a kind that triggers the activation of a Task Processor. In the figure, the detected book is pending of being returned, thus, the Task Processor in charge of the return is activated. Other Task Processors that are associated with the Publication digital object are also activated. In this way, we select the functionality that is relevant for the user given the business context and the physical environment.

The introduced components represent the building blocks that compose any system based on Parkour. The next section introduces usage guidelines for combining these components in the development of Parkour-based solutions.

### 3.2. Programming Model

Having defined this technology-independent architecture, we now define how this architecture can be used from the software developer's perspective. Since we have followed a pluggable model for the architecture, building a system consists in the assembling of the required plug-ins together. Thus, the construction of a Parkour-based solution consists in (1) determining the plug-ins to be developed—or reused if they are already available—and (2) defining how they are integrated in the platform.

In the current architecture, the Task Manager and the Controller Component are generic; therefore, they can be used in any domain. For determining the rest of the components, the analysis of the business process is required. The following aspects should be determined: the tasks that compose the business process, the ID-sensing technologies required for identifying the physical elements that are involved in the process, and the information of interest for these objects. Each of these aspects is supported by a different kind of plug-in in Parkour. A Task Processor is developed to support each of the tasks that require user intervention. Identification Components are developed for the support of ID-sensing technologies. Finally, Data Providers are defined for each information repository required.

Each Parkour plug-in includes some descriptive information that determines how it is integrated in the platform. The Controller Component uses this information to determine the behaviour of the system when multiple plug-ins coexist. This information is expressed by means of a set of attributes that are specific for each kind of component as described below.

Task Processors are normally activated by the Controller Component as a response to some existing pending task. However, it is also possible to activate them for starting new tasks. In this case, the presence of a pending task is not required for offering functionality of the Task Processor to the user. A Task Processor is considered an initiator if it can be activated at any moment for the creation of a new piece of work. In the library example, the return book task can only be accessed if the involved book is pending return. However, the book borrowing task is not performed in response to any pending work; it is performed by user initiative. Thus, the book borrowing Task Processor should be qualified as initiator.

In addition, Task Processors can provide a user interface or not provide one. When a Task Processor is qualified as silent, the actions required for carrying out a task are performed automatically without demanding interaction with the user. Silent Task Processors can access the physical environment in an unobtrusive manner without disturbing the user. Task Processors can also be qualified as requiring confirmation for their activation or not requiring it. In the case of requiring confirmation, the user is informed about the tasks that can be performed on a physical element and he/she explicitly accesses the desired functionality.

Identification Components are classified as capturers or minters according to their function. Capturers are in charge of transferring identifiers from the physical space to the digital one—e.g., a barcode reader. Conversely, Minters are in charge of transferring identifiers from the digital space to the physical one—e.g., a barcode printer. In this way, depending on the needs of the task—detecting or producing identifiers—only the relevant components are considered by the platform. In addition, Identification Components indicate the technologies that they support. When locating Identification Components, the search can be constrained to a specific technology for tasks where the identification mechanism to be used is already known.

Data Providers provide digital information associated with some physical elements that correspond to a given perspective. Data Providers indicate the namespace—*i.e.*, a unique name for the particular view they are providing—for which they offer their services. In this way, collisions are avoided when multiple Data Providers are present. In the case of multiple Data Providers covering the same namespace, all of them are queried until one of them provides results.

### 3.3. Technology Mapping

Having defined the technology-independent concepts that conform the architecture and the programming model that defines how to use this architecture, we now explain the technology mapping. The technology mapping defines how artifacts from the programming model are mapped to a particular technology. More detail about the target technology selected and how architectural concepts can be mapped to the technological solution is provided below.

#### 3.3.1. Target Technology

Since we want to be able to deploy Pesto in a great variety of mobile devices, we have chosen the Java platform for its implementation. The requirements to be fulfilled in the development of Parkour were (1) to offer adequate user interaction in mobile devices, (2) to define a pluggable extension model, and (3) to integrate different data sources. More detail about the technologies used to cover these aspects is provided below:

##### User Interaction

For the definition of user interfaces that can be supported by a wide range of different devices such as mobile devices, desktop computers or TVs, we use JavaFX. JavaFX provides a declarative language for the definition of rich user interfaces based on a scene graph model that supports effects, arbitrary transforms, and animation. Facilities for the definition of triggers and data binding mechanisms make the user interface more responsive to changes in the data. Since it is a client-side technology, it provides an easy access to the local resources of the mobile device. Facilities for accessing remote services are also provided.

##### Extension Model

The Parkour extension model enables the customization of the platform to specific needs. We make use of the Java Plug-in Framework (JPF) to support the plug-in management. JPF provides a runtime engine that dynamically discovers and loads plug-ins. A plug-in is a software component—including Java classes and other resources—that describes itself by means of an XML file. Plug-ins can declare extension points for other plug-ins to extend them. JPF maintains a registry of available plug-ins and the functions they provide.

##### Data Abstraction

For data handling, Service Data Objects (SDO) are used. SDO provides a unified API for handling typed and untyped data, which makes it possible to hide the back-end data source, since it offers homogeneous access for XML, relational or file-based data sources, and others. In addition, SDO permits disconnected data access patterns with an optimistic concurrency control model. These properties are also adequate for the application domain being considered, since data portions from many sources can be combined and updated.

#### 3.3.2. Mapping to a Technological Solution

Having selected target technologies, we now define how the technology-independent concepts described in Section 3.1 are mapped to implementation assets.

##### Task Manager

This component is implemented in Java. It is a repository for pending tasks. Query capabilities are provided thanks to the XPath support that SDO offers. Pending tasks are formed by a header that includes some metadata—reception date, user group in charge of completing the task, *etc.*—and the payload—domain-specific data that the Task Processor will handle. For example, the following XPath expression retrieves a set of tasks that fulfill certain conditions:

//task[type=“org.example.library.return_book” payload/example:Book[@id=“BK31415”]]


In this case, only tasks of the “Return book” type—which is identified by the “org.example.library.return_book” string—are considered. In addition, the payload information is used to filter the tasks in order to retrieve the tasks where a specific book is involved.

##### Task Processors

These components are implemented in JavaFX. Instructions for completing the task and mechanisms for user interaction are provided. The SDO API is used to compose the resulting message that is returned when the task is completed. To access the physical environment, the Controller Component can be queried for Identification Components that fulfill a given criterion. The queries can be adjusted to match the descriptive information provided with the Identification Component plug-ins—their minting or capturing function and the technologies supported.

##### Identification Components

In the implementation of these components, Java has been used to access the ID-sensing functionality, and JavaFX has been used to present their functionality and configuration options to the user. However, some technological bridges may be required for accessing ID-sensing technologies, depending on the drivers available for these technologies. For example, the functionality of a device that lacks Java libraries can be offered as Web Services that can be consumed by Java clients.

##### Data Providers

Data Providers are implemented in Java. In our current implementation, the pieces of information are represented by means of SDO DataObjects. In this way, data with different structures can be manipulated and queried in a homogeneous manner. The structure of the information is defined by means of XML Schema, which is imported at run-time by means of the SDO API.

##### Controller Component

The Controller Component is implemented by means of different Java and JavaFX classes. JavaFX is used to define the user interface parts that are generic for any Parkour application. Java is used for plug-in management by means of the JPF API. Extension points are declared in this component for extending the platform with new Task Processors, Identification Components and Data Providers.

**Figure 3.**
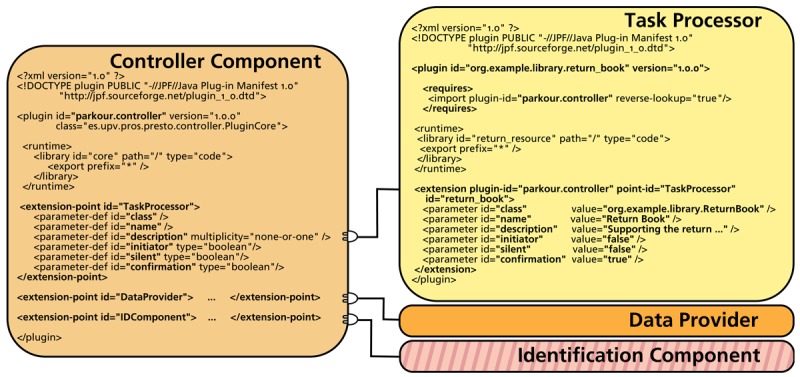
Plug-in descriptors.

Figure 3 shows the plug-in descriptor of the Controller Component plug-in. It includes the declaration of the different extension points and the information that any plug-in that extends them must provide. This figure also illustrates the descriptor for a Task Processor plug-in that extends the platform with support for the return of books task for the library scenario. It is worth noting that the attributes provided by the extender plug-ins (e.g., initiator, silent, confirmation) correspond to the ones introduced in Section 3.2.

As a result, a Java-based implementation for the Parkour platform is obtained. Furthermore, since the concepts defined for the architecture in Section 3.1 and the possibilities for combining them described in Section 3.2 are independent of the technology, the architecture can be easily translated to a different target technology if technological requirements change.

### 3.4. Mock Platform

Having decided the technology for the architecture, we now define a mock platform for developers. With a mock platform, developers can run tests locally as early as possible. The choice of a plug-in infrastructure and SDO as target technologies offers good properties to be used as a mock platform.

A plug-in infrastructure allows components to be easily interchangeable as long as the replacing component provides the same interface as the replaced one. This allows for an easy definition of mock components for test purposes. A component with limited functionality can be used first, and then it can be replaced with a component with the complete functionality.

At the data level, SDO makes it possible to hide the back-end data source, since it offers homogeneous access for XML, relational or file-based data sources, and others. This allows a seamless migration from a mock platform for testing (e.g., based on static XML files) to a production environment (e.g, based on Web Services).

### 3.5. Vertical Prototype

To validate this architecture, we have implemented an application for the resource management of the Department of Information Systems and Computation (DSIC) at the Universidad Politécnica de Valencia. This department plans to adopt ID-sensing to make the lending of different resources (such as laptops, multimedia devices, *etc.*) to the department members more fluent. A prototype of this system has been developed and validated by the department technicians.

In the development of the prototype, we followed the guidelines for infrastructure design and evaluation defined by Edwards [11]. According to these guidelines, we focused on the core infrastructure features, leaving out secondary aspects. While secondary features such as security, distribution, replication and so on are all key to certain scenarios and even necessary for a “real world” deployment, they are not central to the value that Parkour promised to deliver. Thus, we focused on the applicability of the architectural concepts of Parkour and its operation modes. In particular, we evaluated to which extent the platform extensibility and the flexibility in the physical-virtual linkage offered by Parkour improved the support for a subset of the resource management business process. Only three tasks of the process—namely resource creation, resource lend and resource return—are supported. The process includes more tasks such as the resource reservation, but the ones selected are the ones that involve more physical elements.

For each task, a Task Processor was developed as a new plug-in. A user interface is defined for the lend resource task, while the return resource behaves silently and uses the notification mechanism to confirm that the selected resource has been successfully returned. The Task Processor corresponding to the resource creation task, which is in charge of labelling new resources and is qualified as an initiator since this maintenance task can be started at any moment.

Department resources were labeled with QR Codes for their identification. Two Identification Components were developed to support the prototype. One provides access to image capturing devices and wraps the ZXing library for decoding QR Codes. ZXing provides decoding capabilities for QR Codes and other barcode systems. We chose this library since it is open source and is available for different devices—supporting the standard and mobile editions of Java, the Android platform and iPhone. Another Identification Component was developed to cover the minter function. The Google Chart API was used for the production of tags. This API provides support for the generation of QR Code tags. In order to make the recognition as fluent as possible, we selected the “H” error correction level, which is the highest correction level defined by the QR Code standard—it allows the recognition of a code that is damaged up to a 30%.

A *Data Provider* was implemented to transform the obtained identifier into the data object containing the information related to the resource. A static XML file stores the resource information for the developed prototype. Thanks to the use of SDO, the connection to the information provided by the DSIC web services is straightforward.

**Figure 4.**
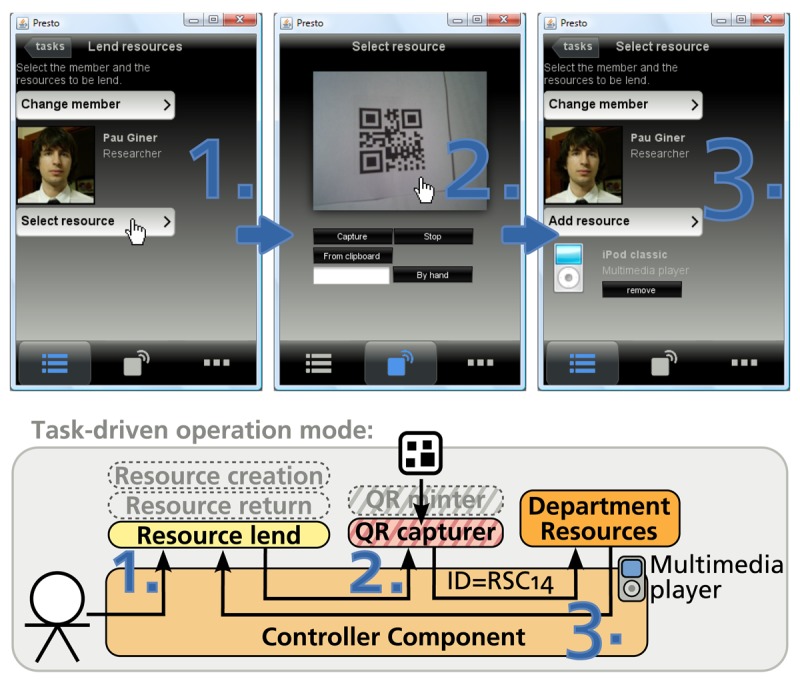
Parkour-based prototype for the resource management in a department.

Figure 4 shows some screenshots of the developed prototype. At the top of the figure, the lending of a resource—a multimedia player—to a department member is shown. At the bottom of the figure, the exchange of information among the different components of the architecture is illustrated. The task-driven operation mode is followed in the example: (1) the user explicitly selects the task to be performed; (2) the user provides information by means of some Identification Component; and (3) the captured identifiers are translated into relevant information by a Data Provider. In contrast, if the object-driven mode were used, the step 1 in the figure would be avoided, since the Controller Component would be in charge of locating the Task Processors that are capable of handling the detected element.

The prototype was deployed in a Samsung Q1 Ultramobile PC, making use of its integrated camera for tag recognition. The department technicians who are in charge of the resource management evaluated the prototype. The two operation modes were used for the lending, returning and creating resources. Based on the prototype evaluation results, the technicians considered that the operation modes offered by Parkour—task-driven and object-driven—were natural for completing their tasks. They perceived an improvement in the business process efficiency in comparison with the web application they used for this task—which was accessed from a desktop computer. Although the prototype performance was affected by the resource limitations of the mobile device, the use of Parkour alleviated the technicians' burden of constantly correlating information between physical and digital spaces, making the process more fluent.

## 4. Consolidation of the Architecture

In order to validate the architecture concepts, better define the programming model and adjust the technology mapping, we have developed Parkour-based solutions to support different case studies. Some of these case studies were considered in a previous work [12] for defining the integration of ID-sensing mechanisms with a business process execution engine. These previously developed systems control the process flow. Parkour complements them by providing the mechanisms to enable users to participate in these processes from their mobile devices. In addition, the Smart Library case study that is used throughout this paper for illustration purposes is also detailed.

### 4.1. Smart Toolbox

The Smart Toolbox [13] case study consists of monitoring the tools used for the process of aircraft Maintenance, Repair, and Overhaul (MRO). During this process, the system should prevent tools from being lost and causing potential damage. To do this, ID-sensing mechanisms are applied to sense the content, the location and the mechanic of each toolbox.

Each step in the MRO process—e.g., preparing the toolbox, performing maintenance and repair tasks, *etc.*—requires a specific functionality for the mechanics. The flexibility in the physical-virtual linkage necessary for this scenario is achieved by providing at each moment the information that the mechanic requires according to the business context. In order to do this, a Task Processor plug-in was developed to support each task in which the mechanics are involved. When a mechanic is preparing the toolbox, he/she is provided (1) a list of the tools that are required for the planned repairs, and (2) services for ordering new tools. For the repair tasks, mechanics can use their mobile devices to retrieve a list of the repair actions to be taken for a particular plane. Once the plane tag is detected by the mobile device, it indicates the specific repairs required and the general maintenance protocol.

The prototype was developed making use of the reacTIVision framework for the real-time detection of specially designed 2D barcodes using a video camera. The reacTIVision framework allows the detection of multiple tags at the same time. This permits the fast detection of several tools in a toolbox. However, the need for a direct line-of-sight required when dealing with 2D barcodes slightly limits the performance of the process. To cover this limitation, a new Identification Component plug-in was developed that supports RFID technology, making the process more fluid. This plug-in wraps the specific libraries of a Caen A828DKEU RFID reader. Thanks to the extensibility of the platform, the technological migration was achieved just by replacing the old plug-in with the new one.

### 4.2. Incidence Management Process

This case study, which was introduced in [14], defines a business process that describes the protocol followed by a company in order to manage the infrastructure incidences reported by company members. It defines both the set of activities that need to be performed and how these are organized among the different participant roles. In this case, the involved roles are the members, the infrastructure manager and the technicians. We modified the original case study to consider that (1) department members are identified by an identity card and (2) material requests are labeled by the provider.

For example, when something is broken, it is reported by a company member. Then, the infrastructure manager contacts the provider to get the required materials to fix the incidence. Finally, members of the technical staff are in charge of the repair. ID-sensing mechanisms are used for the tasks where requested materials and members participate. Since material reception and incidence repair can be separated in time, support is provided for locating the material and indicating where the repair should be performed. Each of these tasks is implemented by a different Task Processor plug-in.

Two Data Provider plug-ins were developed to achieve the required flexibility in the physical-virtual linkage. For each material, the infrastructure manager and the material provider are interested in different aspects. Thus, different Data Providers are developed to support each view. Since materials are tagged with RFID tags, the Identification Component already developed for the Smart Toolbox scenario was used for their identification. In this way, development effort is reduced by leveraging the extensibility of the platform.

Since much of the process correlation was previously performed manually, relying on human memory or e-mail mining for matching orders and materials, the use of ID-sensing improved the process performance. Technicians can access relevant information closer to where the process activities take place.

### 4.3. Smart Library

We also applied Parkour to support business processes in a library. This case study is used throughout the paper for illustration purposes. The extensibility of the platform enabled the creation of custom applications for the different business process participants just by combining plug-ins. The mobile client for the library members includes a Task Processor plug-in for each of the tasks they perform in the process—e.g., borrowing a book and comment on books—as well as an Identification Component plug-in to support QR Code-based identification. Librarians make use of the Identification Component that supports RFID technology for supporting a different set of tasks—e.g., notify return of books and place them to their shelf.

The book lending process is complemented by services enabling members to share book-related comments. While book lending concerns books at the item level, comments are not specific to the book itself but rather to its content. Thus, two different Data Provider plug-ins were defined to represent these views in order to offer flexibility in the physical-virtual linkage.

**Figure 5.**
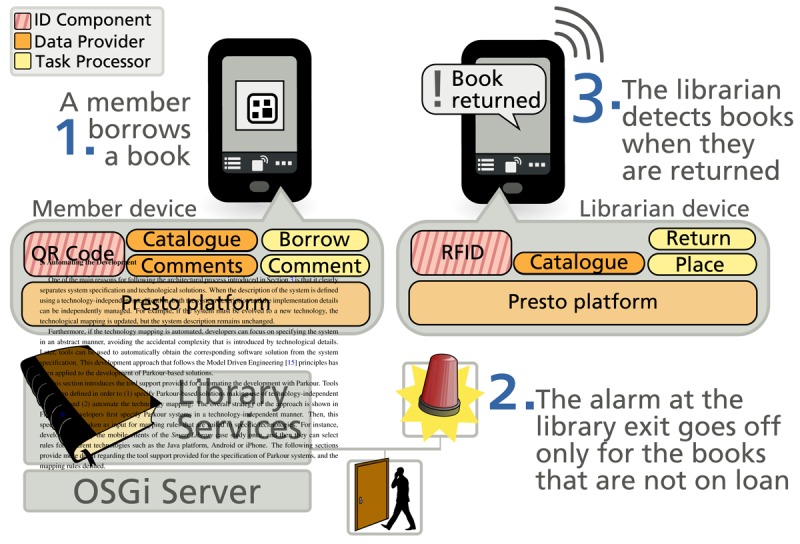
Set-up for the Smart Library scenario.

Figure 5 shows an overview of the library scenario. Two mobile devices interact with the library Information System. Both clients are based on the Parkour platform but they extend it with specific plug-ins. These plug-ins support the specific perspective of each participant in the process. The library services are based on OSGi and we make use of KNX devices to support the detection of books at the exit of the library and the alarm system in a scale environment. An Ultramobile PC that integrates two cameras was used as a mobile device for users. The WiFi capabilities of the Ultamobile PC were used for its integration with the library services.

## 5. Automatic the Development

One of the main reasons for following the architectural process introduced in Section 3 is that it clearly separates system specification and technological solutions. When the description of the system is defined using a technology-independent specification, both the system description and the implementation details can be independently managed. For example, if the system must be evolved to a new technology, the technological mapping is updated, but the system description remains unchanged.

Furthermore, if the technology mapping is automated, developers can focus on specifying the system in an abstract manner, avoiding the accidental complexity that is introduced by technological details. Later, tools can be used to automatically obtain the corresponding software solution from the system specification. This development approach that follows the Model Driven Engineering [15] principles has been applied to the development of Parkour-based solutions.

**Figure 6.**
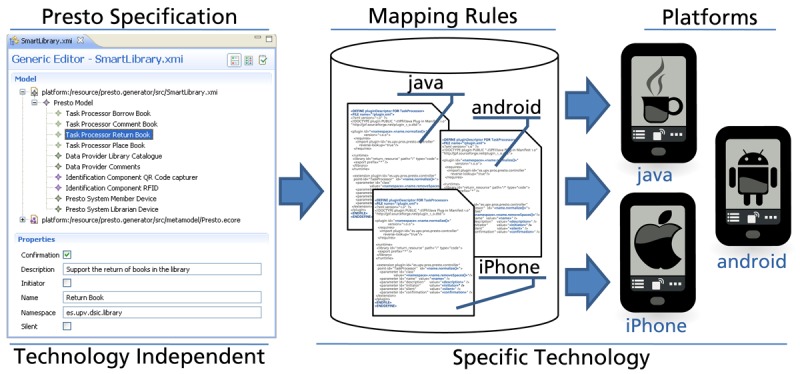
Automating the Development.

This section introduces the tool support provided for automating the development with Parkour. Tools have been defined in order to (1) specify Parkour-based solutions making use of technology-independent concepts, and (2) automate the technology mapping. The overall strategy of the approach is shown in Figure 6. Developers first specify Parkour systems in a technology-independent manner. Then, this specification is taken as input for mapping rules that are suited to specific technologies. For instance, developers specify the mobile clients of the *Smart Library* case study once, and then they can select rules for different technologies such as the Java platform, Android or iPhone. The following sections provide more detail regarding the tool support provided for the specification of Parkour systems, and the mapping rules defined.

### 5.1. Technology-Independent Specifications

We provide tools for supporting the definition of technology-independent descriptions (*i.e.*, models) of Parkour systems. The concepts introduced in the technology-independent architecture defined in Section 3.1 are used as building blocks for specifying Parkour models. In this way, developers avoid to deal with the particularities of the target technology selected for Parkour implementation.

In order to provide tool support for specifying Parkour models, the technology-independent architecture has been formalized using an Ecore metamodel. A metamodel captures the constructs that can be used to describe systems and the ways in which these constructs can be combined. For example, the Parkour metamodel determines that a Parkour-based system is composed by several Task Processors, each of them containing attributes such as name, initiator, silent, *etc.* Ecore, which is part of the Eclipse Modeling Framework [16] (EMF), provides support for the definition of structured specifications with precise semantics. EMF facilites the definition of editors for system specifications that are based on Ecore metamodels. Figure 6 (left) shows an EMF-based editor for Parkour systems.

We have defined the Parkour metamodel as the first step towards the automation of the development process. The use of EMF enables metamodels to be machine-processable. This allows other EMF-compliant tools to manipulate Parkour specifications with different purposes—check properties, define graphical editors for the specification, *etc.* Thus, Parkour becomes also an extensible platform at tool level. In particular, we make use of code generation techniques in this work to automate the technology mapping as it is illustrated in next section.

### 5.2. Automating the Technology Mapping

The technology mapping introduced in Section 3.3 involves several repetitive tasks. For our target technology, the definition of each *Task Processor* involves actions like the definition of a JavaFX class that extends the *Task Processor* interface and the definition of the XML description file for each plug-in following the JPF format. This boilerplate code can be automatically generated from the information captured in Parkour models.

To automate the technological mapping, we have defined rules that describe how one or more concepts in Parkour can be transformed into one or more concepts in the target technology. We have developed these mapping rules by means of the openArchitectureWare (oAW) toolset. We have defined mapping rules for the generation of the software artifacts that are required to support a Parkour-based system for the target technology selected in this work. In particular, from a Parkour specification, the complete plug-in descriptors and code skeletons for the required Java and JavaFX assets are generated. Developers must complete the generated skeletons with the application-specific functionality. Although a fully functional system is not obtained by the mapping rules, the produced assets are useful to guide the development. In this way, developers can focus on the business logic and avoid dealing with infrastructure aspects such as the particular format that JPF uses for plug-in descriptors.

**Figure 7.**
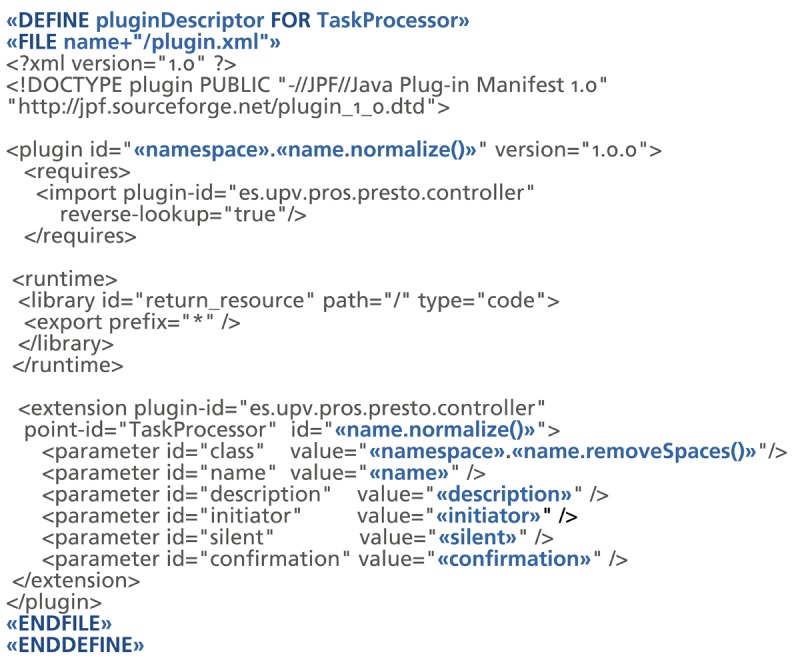
Example of mapping rule.

Figure 7 shows the mapping rule defined for the generation of *Task Processor* plug-in descriptors. The rule named *pluginDescriptor* is defined for the *Task Processor* element from the Parkour metamodel. The *FILE* statement defines the output file for the code generation—in this case a plugin.xml file is generated into a folder named after the Task Processor.

The rest of the rule is a code template with static and dynamic parts. The static pats of code are transferred to the generated code directly. In the example template, the static parts represent aspects that are common to any Task Processor plug-in such as the plug-in header—see the plug-in descriptor from Figure 3 as an example. The dynamic code is calculated for each instance to which the rule is applied. Dynamic expressions—defined between angle quotes—are used for capturing the required information and expressing it according to the target technology. For example, the ≪ *name.normalize* ≫ expression applies the normalize function—which replaces whitespaces with underscores—to the Task Processor name string in order to obtain an identifier for the plug-in that is valid according to JPF.

The mapping rules defined in the present work provide support to generate code for our target technology based on the Java platform. However, the catalogue of mapping rules can be extended in order to translate the Parkour concepts into other mobile platforms.

## 6. Related Work

Many of the clients supporting object hyperlinking are either generic or specific for a given task. On the one hand, the generic clients process tags in the same way according to different protocols that are indicated in the tag, e.g., accessing a given URL. This involves coupling the identification with the associated service [8]. This coupling makes it impossible to provide new services for an object without altering the tag—something that is costly when a great number of elements are involved or the elements are out of the physical control of the organization as in post-sale services. Conversely, our approach promotes the decoupling between identifiers and services in order to support the different perspectives that each participant in a business process requires. On the other hand, specific clients normally represent ad-hoc solutions that are coupled to a specific technology and the effort in their development is difficult to reuse. The extensible nature of our approach allows to add specific processors that handle the detected tags in different ways and it also allows to incorporate new ID-sensing technologies as they appear.

Several research initiatives have had the goal of reducing the physical-virtual gap in business processes. However, much of the effort is focused on automating processes, and little attention has been paid to how to facilitate user participation. Wieland defines the Smart Workflows [1] concept and provides an architecture for transforming the low-level data that is captured by different kinds of sensors—not only those based on ID-sensing—into information at the business level. Other infrastructure solutions [12,17,18] specifically focus on integrating ID-sensing with business process management solutions. In contrast, in Parkour, users have a principal role in the interaction with the environment and the management of their business contexts. Involving the user in these aspects limits the level of automation for the process, but it makes users feel that they are in control of their activities. Previous studies confirm the benefits provided by the explicit user interaction by means of the ID-sensing capabilities of mobile devices [8,19] and the user-centered management of context-awareness in mobile computing [20].

Workflow technologies have been also applied to integrate sensor information of different kinds. Business process engines have been effective when used as part of the infrastructure to process large volumes of Sensor Web data to feed geospatial services [21]. A RESTFul based workflow interoperation is proposed at [22] to structure a workflow that accesses large-scale sensor information in an automated fashion. Another approach based on SOA technologies is provided at [23]. These works are mainly focused on interoperability, flexibility, reusability, and high performance. Our approach is focused on the support of workflows with human intervention to serve the user needs better at each point of the process. Nevertheless, our approach would benefit from integrating the data collection approaches described above and exposing them as Data Providers, since more data and more accurate information about the user surroundings represent new opportunities to better serve the user needs.

Another aspect of interest for this work is the use of mobile devices. Research in mobile business processes—*i.e.*, specific kinds of business processes where most of the human interaction is performed using mobile devices—is mainly focused on providing technological support for process execution. Execution engines for mobile devices that are based on WS-BPEL have been developed [24,25]. These engines have been designed to overcome the limitations of WS-BPEL when it is used for supporting the dynamic nature of ubiquitous services—e.g., enabling the dynamic change of participants. Since these two works are focused on process coordination, they do not provide mechanisms for the integration of physical elements. However, the integration of these proposals with Parkour can be interesting since they deal with different but complementary aspects such as process orchestration and user participation. This integration could be especially interesting in the case of [25] since it makes use of EMF tools to support the modeling and code generation for their platform, which makes it possible to inter-operate with Parkour at tool level.

## 7. Conclusions and Further Work

This work defines a platform for supporting user participation in smart workflows. We do not consider this as a one-size-fits-all solution, but rather a solution that can be adapted to the specific needs of a business process. The extensibility of the platform and the flexibility of the physical-virtual linkage make it possible to offer users the functionality that they require according to their role in the process.

The architectural process followed in this work decouples the architectural concepts from the particular technological choices, improving the evolution capabilities of the resulting systems. The architecture has been used in several case studies and proven suitable for the automation of the development. In addition, a version for Android is currently under development, showing that the architectural concepts are translatable to different technologies. The integration of our platform with tools that are familiar to business analysts such as BPMN diagram editors would permit the development of Smart Workflows to be dealt with in a declarative manner. The development of tools and techniques for achieving this integration constitutes the final goal of this research.
